# Novel Approach to Cell Surface Discrimination Between KIR2DL1 Subtypes and KIR2DS1 Identifies Hierarchies in NK Repertoire, Education, and Tolerance

**DOI:** 10.3389/fimmu.2019.00734

**Published:** 2019-04-05

**Authors:** Jean-Benoît Le Luduec, Jeanette E. Boudreau, Julian C. Freiberg, Katharine C. Hsu

**Affiliations:** ^1^Immunology Program, Memorial Sloan Kettering Cancer Center, Sloan Kettering Institute, New York, NY, United States; ^2^Department of Medicine, Memorial Sloan Kettering Cancer Center, New York, NY, United States; ^3^Weill Cornell Medical College, New York, NY, United States

**Keywords:** KIR2DL1, KIR2DS1, NK cell, NK cell education, HLA-C2, KIR allele

## Abstract

Cumulative activating and inhibitory receptor signaling controls the functional output of individual natural killer (NK) cells. Investigation of how competing signals impact response, however, has been hampered by the lack of available antibodies capable of distinguishing inhibitory and activating receptors with highly homologous ectodomains. Utilizing a novel combination of monoclonal antibodies with specificity for discrete inhibitory KIR2DL1 and activating KIR2DS1 allotypes found among 230 healthy donors, we investigated allele-specific receptor expression and function driven by *KIR2DL1* and *KIR2DS1* alleles. We found that co-expression of the HLA-C2 ligand diminishes KIR2DL1, but not KIR2DS1, cell surface staining, but does not impact the respective frequencies of KIR2DL1- and KIR2DS1-expressing cells within the NK repertoire. We can distinguish by flow cytometry NK cell populations expressing the most common KIR2DL1-C^245^ allotypes from those expressing the most common KIR2DL1-R^245^ allotypes, and we show that the informative differential binding anti-KIR2DL1/S1 clone 1127B is determined by amino acid residue T^154^. Although both KIR2DL1-C^245^ and KIR2DL1-R^245^ subtypes can be co-expressed in the same cell, NK cells preferentially express one or the other. Cells expressing KIR2DL1-C^245^ exhibited a lower KIR2DL1 cell surface density and lower missing-self reactivity in comparison to cells expressing KIR2DL1-R^245^. We found no difference, however, in sensitivity to inhibition or cell surface stability between the two KIR2DL1 isoforms, and both demonstrated similar expansion among NKG2C^+^ KIR2DL1^+^ NK cells in HCMV-seropositive healthy individuals. In addition to cell surface density of KIR2DL1, copy number of cognate *HLA-C2* hierarchically impacted the effector capacity of both KIR2DL1^+^ cells and the tolerization of KIR2DS1^+^ NK cells*. HLA-C2* tolerization of KIR2DS1^+^ NK cells could be overridden, however, by education via co-expressed self-specific inhibitory receptors, such as the heterodimer CD94/NKG2A. Our results demonstrate that effector function of NK cells expressing KIR2DL1 or KIR2DS1 is highly influenced by genetic variability and is calibrated by co-expression of additional NK receptors and cognate HLA-C2 ligands. These findings define the molecular conditions under which NK cells are activated or inhibited, potentially informing selection of donors for adoptive NK therapies.

## Introduction

NK cells are key innate lymphocytes, capable of discriminating malignant or infected cells from their healthy counterparts. They are controlled by a multiplicity of combinations of inhibitory and activating receptors, including the killer immunoglobulin-like receptors (KIR). Inhibitory KIR and the heterodimer CD94/NKG2A (NKG2A) recognize self-human leukocyte antigen (HLA) class I molecules, whose presence on healthy cells protects from NK autoaggression, but whose absence on diseased cells permits NK release of cytotoxic granules, resulting in targeted cell killing (1). The same interaction between the inhibitory receptor and its cognate HLA class I ligand in the tissue environment lowers the threshold for functional responses in a process termed “education,” “licensing,” or “tuning” (2), resulting in an NK repertoire in which cells expressing inhibitory receptors for self-MHC exhibit high functional and killing capacity when encountering MHC-deficient, damaged cells, but are inhibited from killing HLA-sufficient healthy cells. In contrast, NK cells expressing inhibitory receptors for which the individual lacks the cognate HLA class I ligand are relatively hyporesponsive, thereby avoiding potential autoreactivity. Activating KIR are less well-characterized, but can recognize classical or non-classical class-I HLA proteins and drive cellular activation or tolerance to self (3–5). Recent studies have demonstrated peptide specificity for various activating KIR (6–8).

The inhibitory KIR2DL1 and activating KIR2DS1 receptors have highly homologous extracellular domains (9), and both bind HLA-C allotypes with Lys80, collectively referred to as group *HLA-C2* alleles. Signaling opposite effects upon engagement with the same HLA ligand, KIR2DL1 endows the NK cell with functional competence but inhibits the NK cell when encountering HLA-C2 on neighboring cells, while KIR2DS1 signals activation and cytotoxicity toward the same cell. In yet another aspect of NK education, KIR2DS1-bearing NK cells in individuals homozygous for *HLA-C2/C2* are tolerized to the ligand on surrounding cells, thereby avoiding autoreactivity (4, 5).

Various KIR-HLA interactions influence NK education with known impacts on human health (3). Subtype variability for KIR3DL1 and its ligand HLA-Bw4 diversifies NK cell response, with predictable impacts on the outcome of hematopoietic cell transplantation in patients with leukemia, antibody therapy in patients with neuroblastoma, and killing of HIV-infected autologous CD4^+^ T cells (10–12). *KIR2DS1* in hematopoietic cell donors is beneficial to *HLA*-matched recipients, but only in the absence of homozygosity for *HLA-C2*, which renders KIR2DS1^+^ NK cells hyporesponsive (13). Finally, the *KIR2DL1* alleles associated with *KIR-A*, but not *KIR-B* haplotypes have been recently shown as positively correlated with the likelihood of developing pre-eclampsia (14). The majority of studies have investigated the impacts of single *KIR-HLA* partnerships in isolation, but, in reality, the majority of NK cells express more than one receptor that can interact with HLA or other ligands; understanding how this diversity impacts outcomes will therefore be a critical step toward fully understanding NK cell interactions and potential function against diseased cells.

To date, 38 different alleles have been described for *KIR2DL1* and nine alleles for KIR2DS1 (15). Previous studies have demonstrated that copy number and allelic variation of inhibitory *KIR* impact frequency of receptor expression in the NK repertoire and density on the cell surface (16–18). Only dimorphism of the amino acid in position 245 [arginine (R) or cysteine (C)] of KIR2DL1 has been shown to have a functional impact, with KIR2DL1-C^245^ allotypes demonstrating lower capacity for inhibition against cognate HLA (16). However, this study was completed using cell line transfectants; whether the same dimorphism is relevant in primary human NK cells has not been tested. Conducting these studies has been challenging, due to lack of high throughput technologies to identify *KIR* alleles routinely (19–21) and access to ethnically diverse populations bearing allelic variability in the KIR genes of interest. Lack of antibodies that can distinguish between the highly homologous inhibitory and activating KIR2DL1/S1 isoforms and their allele subtypes further hampered the ability to discern the contribution of each receptor to NK cells bearing both.

We recently developed a method to distinguish *KIR2DL* alleles and allele groups and genotyped a bank of PBMC from 230 ethnically diverse healthy individuals (22). In the present study, we investigate how KIR2DL1/S1 allelic diversity, allele copy number, and co-expression of the HLA-C2 ligand impact the NK repertoire and education in individuals without a large human cytomegalovirus (HCMV)-associated adaptive NK cell compartment, which can skew the NK repertoire toward a more educated population (17, 23). Using a novel combination of antibodies with known specificities for KIR2DL1 allotypes, we can now compare allotype-specific differences in KIR2DL1 and KIR2DS1 expression and function on cell populations with discrete combinations of receptor allotypes within the same individual. Our results demonstrate that effector function of NK cells expressing KIR2DL1 or KIR2DS1 is highly influenced by genetic variability and co-expression of HLA-C2 ligands, and demonstrate how multiple inhibitory and activating interactions collaborate for NK cell education at the single cell and repertoire levels. Taken together, these findings endorse study of multiple receptor-ligand partnerships in combination to best predict NK cell function. This understanding of the dynamic and hierarchical contributions of allele and copy number variation, activating and inhibitory input may inform donor selection for hematopoietic stem cell transplant, selection of efficient NK cells in cellular therapy for cancer treatment, and prognosis for patients with infectious disease.

## Methods

### Cell Sources and Preparation

DNA samples were extracted using blood mini kits according to the manufacturer's instructions (Qiagen). Peripheral blood samples were collected from healthy human donors following approval from the Memorial Sloan Kettering Cancer Center (MSKCC) Institutional Review Board, and donors provided informed, written consent. Peripheral blood mononuclear cells (PBMC) were isolated by Ficoll centrifugation. Additional PBMC were isolated from buffy coats obtained from healthy volunteer donors via the New York Blood Center (NYBC, http://nybloodcenter.org/). The MSKCC IRB waived the need for additional research consent for anonymous NYBC samples. PBMC were cryopreserved in fetal bovine serum with 10% DMSO. Human cytomegalovirus (HCMV) serostatus was provided by NYBC.

### HLA-Class I and *KIR2DL1* and *KIR2DS1* Allele Typing and Copy Number Calculation

An intermediate resolution Amplification Refractory Mutation System (ARMS) PCR method for distinguishing *KIR2DL1* allele groups and some individual alleles was used to identify *KIR2DL1* alleles for 230 donors (22). The majority of donor samples used in the present study (200/230) also had allele-level genotyping confirmed by sequence-based typing in a previous study (21, 22). Copy number for *KIR* was imputed based on known linkage disequilibrium between alleles permitting *KIR* haplotype assignment (22, 24). Allele typing for *HLA-A, B*, and *C* was performed by Histogenetics (Ossining, NY), and KIR ligand designations were assigned using the IPD-HLA database Version 3.34.0 (available online: https://www.ebi.ac.uk/ipd/imgt/hla/).

### Target Cells, Effector Cells, and Culture Conditions

The cell lines K562 (ATCC), 721.221, 721.221 transfected with cDNA encoding HLA-Cw2 or HLA-Cw4, collectively referred to as HLA-C2 (kindly provided by Dr. Peter Parham, Stanford University, Stanford, CA), HL60 [HLA-C2/C2, genotype HLA-C^*^06:02/C^*^06:02 (25)] (ATCC) and MONO-MAC-1 (HLA-C1/C2) (kindly provided by Dr. Stephen Nimer, University of Miami, Miami, FL) were used as target cells for *in vitro* assays and were cultured in RPMI-1640 medium supplemented with 10% heat-inactivated fetal bovine serum, 100 U/ml penicillin, 100 μg/ml streptomycin, 1% sodium pyruvate, and 1% 2-mercaptoethanol and incubated at 37°C with 5% CO_2_. To upregulate HLA class I expression, recombinant human IFN-γ (PeproTech) was added at 1,000 units/mL daily for 72 h (12, 26, 27). Effector cells were cultured in medium, described above, supplemented with human IL-2 (Proleukin, Prometheus) at 200 U/mL and incubated at 37°C with 5% CO_2_ for 12 to 16 h prior to assaying function.

### Phenotypic Analysis by Flow Cytometry

PBMCs (2 × 10^5^ cells per well) were stained 25 min at room temperature with the following antibodies: anti-CD56, anti-CD3, anti-CD158a with or without anti-CD158h and with anti-NKG2C (Table 1). Dead cells were excluded by staining with DAPI (Invitrogen), and NK cells were defined by CD3^−^CD56^dim^-gated cells. HLA-C class I expression was evaluated with the anti-HLA-C antibody produced by the DT9 hybridoma (kindly provided by Dr. Mary Carrington, NCI Frederick) with a secondary staining with goat anti-mouse IgG (Ab specifically adsorbed to minimize cross-reactivity). All FACS analyses were performed on an LSR Fortessa (BD Biosciences) and analyzed using FlowJo software (9.8.5, Treestar). For multicolor compensation and gating, unstained, single-color, and FMO controls were used. Donors with NKG2C^+^ expanded NK cells were excluded from studies, except where specifically noted, given that cytomegalovirus infection can modify NK repertoire, leading to stable imprints, which may confound our interpretations (17).

**Table 1:** Antibodies.

**Antibody**	**Clone**	**Color**	**Provider**
anti-CD56	N901	ECD	Beckman Coulter
anti-CD3	UCHT1	BV650	BD Bioscience
anti-CD158a	143211	APC, FITC	R&D systems
anti-CD158h	1127B	PE, APC	R&D systems
anti-CD158a/h	EB6B	PE, PEC5.5	Beckman Coulter
anti-CD158a/h	11PB6	APC, PE	Miltenyi
anti-CD158b1/b2/j	GL183	PEC5.5	Beckman Coulter
anti-CD158b1/b2/j	DX27	APC	Miltenyi
anti-CD158e1	DX9	BV711	BD Biosciences
anti-CD158e1/e2	Z27	APC	Beckman Coulter
anti-CD158i	JJC11.6	FITC	Miltenyi
anti-NKG2A	Z199	PEcy7	Beckman Coulter
anti-NKG2C	134591	Alexa488, Alexa700, APC	R&D systems
anti-HLA-C	DT9	–	Carrington Lab
anti-mouse IgG	Poly4053	APC	Biolegend
anti-CD107a	H4A3	BV786	BD Bioscience

### NK Activation by Flow Cytometric Analysis

CD107a mobilization was used to determine effector cell activation. PBMCs (2–5 × 10^5^ cells per well) were incubated with target cells at a 1:5 ratio in U-bottom 96 well plates in the presence of anti-CD107a. After co-culture, cells were stained with anti-CD56, anti-CD3, anti-CD158a, anti-CD158h, anti-CD158b1/b2/j, anti-CD158i, anti-CD158e1, anti-NKG2A, and anti-NKG2C (Table 1). NK cells exclusively expressing a single inhibitory KIR receptor were evaluated, excluding cells co-expressing other inhibitory receptors that could contribute to NK education (KIR2DL2, KIR2DL3, KIR3DL1, and NKG2A) or activating receptors (NKG2C and KIR2DS2), and cells co-expressing KIR2DL1 and KIR2DS1 could be discriminated using a combination of antibody clones 1127B and 143211 (Table 1), as described in Results. For activation with soluble antibody, PBMC were incubated with a saturating concentration of conjugated 1127B mAb (0.25 μg/mL) or 11PB6 (2.5 μg/mL) mAb prior to co-incubation with target cells.

### DNA Constructs and Transfections

KIR2DL3^*^005 was used as a target for site-directed mutagenesis to determine the critical amino acid residues for 1127B antibody binding. KIR2DL3^*^005 cDNA was cloned into pcDNA3.1(+) (Invitrogen), and point mutations were introduced by substitution with the Q5 Site-Directed Mutagenesis Kit (New England BioLabs) according to the manufacturer's instructions. DNA probes were designed with the online software NEBaseChanger. All constructs were prepared as per manufacturer's instructions using the QIAprep Spin Miniprep Kit (QIAGEN) and transformed into SURE 2 Supercompetent Cells (Stratagene) following the manufacturer's instructions. cDNA sequences were confirmed by Sanger sequencing (MSKCC DNA Sequencing Core Facility). HEK293 cells (ATCC) were transfected using jetPRiME (Polyplus-Transfection) according to the manufacturer's instructions and cultured in DMEM medium supplemented with 10% heat-inactivated fetal bovine serum, 100 U/mL penicillin, 100 μg/mL streptomycin, and incubated at 37°C with 5% CO_2_. Transgene expression was measured by flow cytometry 48 h after transfection.

### Statistics

Unpaired or paired Mann-Whitney tests were used to compute comparisons between the phenotypically different NK populations. A test with a *p* < 0.05 was considered statistically significant.

## Results

### The Frequency of NK Cells Expressing KIR2DL1 or KIR2DS1 Is Predicted by the Specific Allele and by Copy Number, but Not Influenced by the Presence of Cognate Ligand

**Figure 1:**
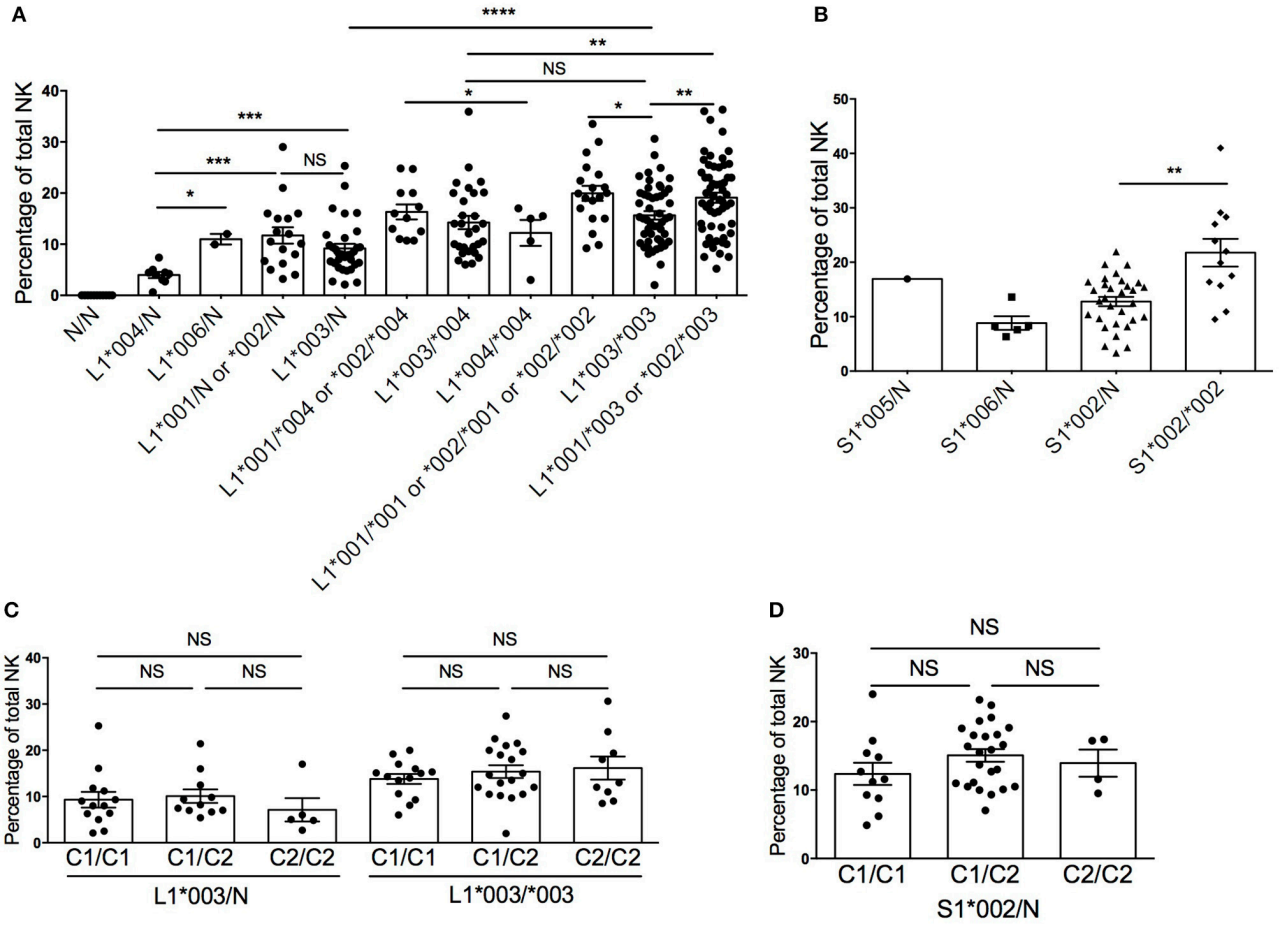
Diversity in frequency of *KIR2DL1*^+^ and *KIR2DS1*^+^ NK cells according to allele typing in 230 healthy blood donors. **(A)** *KIR2DL1* allele/copy number and frequency of KIR2DL1^+^ NK cells. **(B)** *KIR2DS1* allele and copy number and frequency of *KIR2DS1*^+^ NK cells. **(C)** HLA-C KIR ligands and frequency of *KIR2DL1*^+^ NK cells. **(D)** HLA-C KIR ligands and frequency of *KIR2DS1*^+^ NK cells. N represents absence of a KIR2DL1 allele on the KIR haplotype. Unpaired Mann-Whitney test was used. Symbols represent individual samples (mean ± SEM). *p < 0.05, **p < 0.01, ***p < 0.001, and ****p < 0.0001.

Expression of KIR molecules, including KIR2DL1 and KIR2DS1, has been described as “stochastic” or random (28, 29), but more recently, investigations into allele subtype diversity have demonstrated that expression frequencies and cell surface densities of KIR molecules can be predicted by their allele subtype and copy number (10, 18, 30, 31). To determine if the copy number of KIR2DL1 and KIR2DS1 influences the frequency of NK cells expressing the receptors, we stratified 230 healthy human donors based on their *KIR2DL1* and *KIR2DS1* allele typing and allele copy number and measured the frequency of NK cells expressing KIR2DL1 or KIR2DS1 (Figures 1A,B). Consistent with published findings (20), we found the most common alleles for *KIR2DL1* were ^*^002, ^*^003, and ^*^004; for *KIR2DS1*, donors most frequently carried the ^*^002 allele. In general, we found that donors exhibiting two copies of *KIR2DL1* showed higher frequencies of *KIR2DL1*^+^ NK cells in their repertoires. Among KIR2DL1 allotypes, ^*^004 was expressed on the lowest number of NK cells, but KIR2DL1^*^001, ^*^002 and ^*^006 were similarly expressed. Compared with donors exhibiting a single copy of the KIR2DL1^*^003 allele, the frequency of KIR2DL1^+^ NK cells approximately doubled in donors having two copies of the same allele (*p* < 0.0001) (Figure 1A). In donors with two different *KIR2DL1* alleles, the frequency of KIR2DL1^+^ NK cells reflected the additive frequencies of each allele alone. Similarly, the frequency of KIR2DS1^+^ NK cells was lower in donors exhibiting one allele copy compared with those encoding two; this was especially pronounced when we limited our analysis to donors encoding one vs. two allele copies of KIR2DS1^*^002, the most common *KIR2DS1* allele (*p* < 0.01) (Figure 1B).

Previous studies have reported that co-inheritance of HLA ligands can impact the frequency of NK cells exhibiting cognate KIR among human repertoires (18, 32). Our finding that KIR2DL1^+^ and KIR2DS1^+^ NK cell frequencies are impacted by allele subtype, however, suggests an alternate explanation for these findings. Donors exhibiting a large HCMV-associated adaptive NKG2C^bright^ NK cell population were excluded, as these cells have been reported to have modulated functional capacity (33, 34) and skew the NK repertoire toward an educated phenotype (17, 23). Limiting our analysis to the most common alleles for KIR2DL1 (^*^003) and KIR2DS1 (^*^002), and stratifying based on donor copy number for *HLA-C2*, we observed no significant changes in the frequency of KIR2DL1^*^003^+^ or KIR2DS1^*^002^+^ NK cells (Figures 1C,D). Hence, the frequency of KIR2DL1^+^ or KIR2DS1^+^ NK cells among human NK cell repertoires is driven by allele subtype and gene copy number, but not by cognate HLA-C2 ligand.

### Cell Surface Density of KIR2DL1 Is Diminished in the Presence of the Cognate HLA-C2 Ligand and Influenced by Allelic Variation, but Unchanged by Copy Number

**Figure 2:**
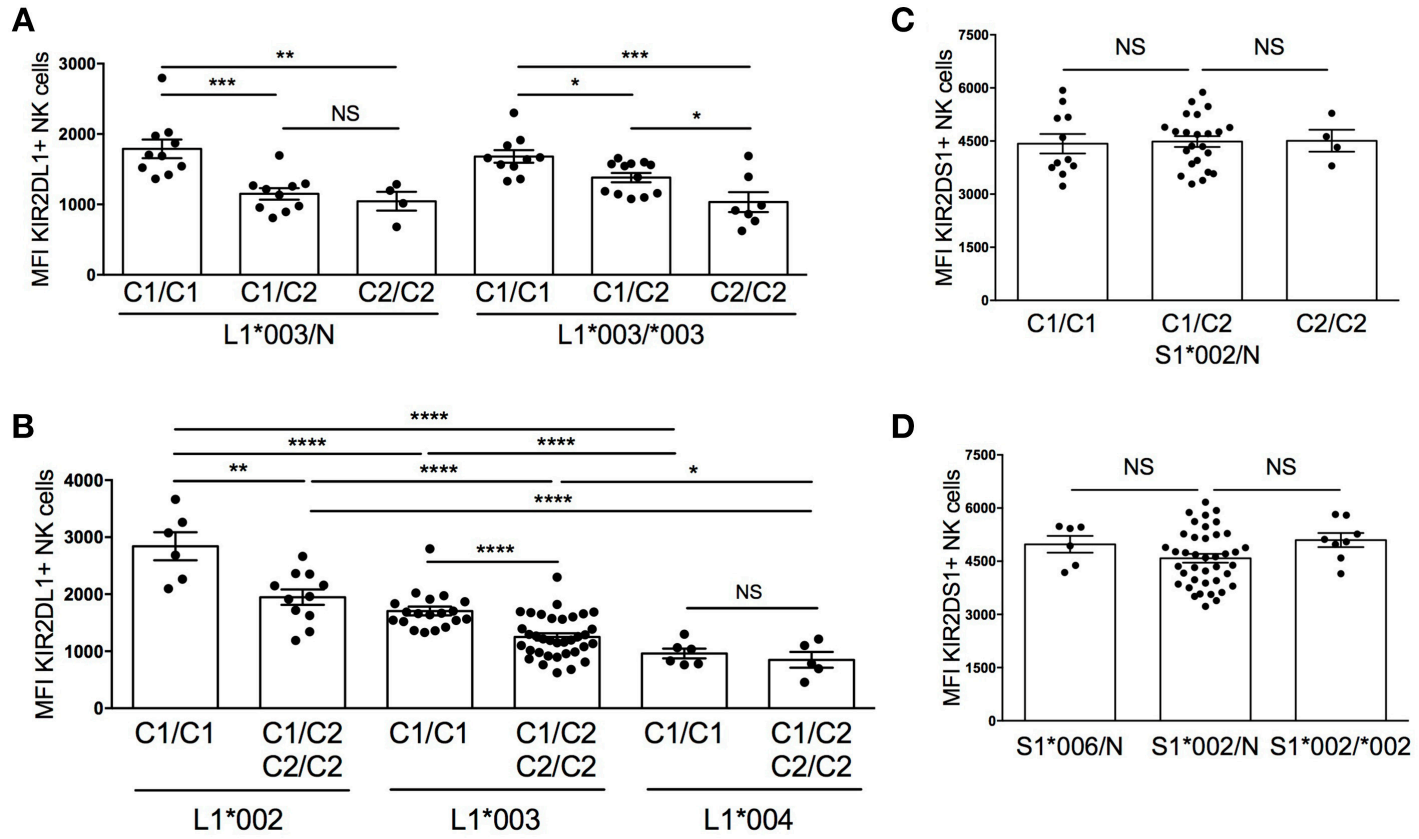
Diversity in cell surface expression of *KIR2DL1* and *KIR2DS1* alleles. KIR2DL1 and KIR2DS1 cell surface expression was measured by flow cytometry on NK cells from healthy blood donors. **(A)** KIR2DL1 cell surface expression segregated by *KIR* allele copy number and HLA-C KIR ligand. **(B)** Diversity of cell surface expression associated with *KIR2DL1* allele typing. **(C)** *KIR2DS1* cell surface expression segregated by KIR ligand. **(D)** *KIR2DS1* allele, copy number, and *KIR2DS1* cell surface expression. Two hundred thirty individuals were evaluated. Unpaired Mann-Whitney test was used. Symbols represent individual samples (mean ± SEM). *p < 0.05, **p < 0.01, ***p < 0.001, and ****p < 0.0001.

A previous study has reported a reduction in cell surface density of KIR when cognate ligands are present in a donor's genome (18). To determine whether this remains true even after *KIR2DL1* and *KIR2DS1* allelic variation are considered, we assessed the median fluorescent intensity (MFI) of KIR2DL1^*^003, KIR2DL1^*^002 and KIR2DL1^*^004 as a measure of receptor cell surface density, stratifying based on the presence and copy number of cognate HLA-C2 molecules. The MFI of KIR2DL1 did not differ based on *KIR* allele copy number, suggesting that only one copy of each gene is active in a given NK cell, and consistent with previous studies that have demonstrated dominant expression of single KIR allotype in individual NK cells (30). However, in comparison to donors who lack the HLA-C2 ligand (*HLA-C1/C1*), KIR2DL1^*^003 MFI was lower on NK cells from individuals expressing one or two copies of *HLA-C2* (*p* < 0.001 and *p* < 0.01, respectively) (Figure 2A).

Since MFI did not change with *KIR2DL1* copy number, we next grouped donors according to *KIR2DL1* allele typing and stratified analysis based on donor *HLA-C2* copy number. Like for KIR2DL1^*^003, we found that expression of HLA-C2 ligands reduced the MFI of KIR2DL1^*^002, measured by the 14321 antibody; however, KIR2DL1^*^004 was found to exhibit low cell surface density that was not significantly influenced by co-expression of cognate HLA-C2 (Figure 2B). In individuals lacking HLA-C2 ligand (*HLA-C1/C1)*, KIR2DL1^*^002 expression exhibited the highest MFI in comparison to ^*^003 and ^*^004 (*p* < 0.0001) and ^*^003 was more densely expressed than ^*^004 (*p* < 0.0001). Similar results were observed between *KIR2DL1* alleles using the anti-CD158a/h EB6B mAb (Supplemental Figure 1), which binds a different epitope than the 14321 antibody, suggesting that expression density differs between isoforms and is not due to differences in the availability of epitopes or binding affinities for each antibody. For KIR2DS1, neither the presence of the cognate ligand HLA-C2, allele copy number nor specific allele impacted MFI (Figures 2C,D). Altogether, these findings suggest that expression of cognate HLA-C2 ligand impacts the availability of high-expressing KIR2DL1 allotypes, with no apparent impact on surface density of the low-expression allotype KIR2DL1^*^004 or of KIR2DS1.

### Antibody Clone 1127B Binds KIR2DL1 Allotypes Exhibiting T^154^

**Figure 3:**
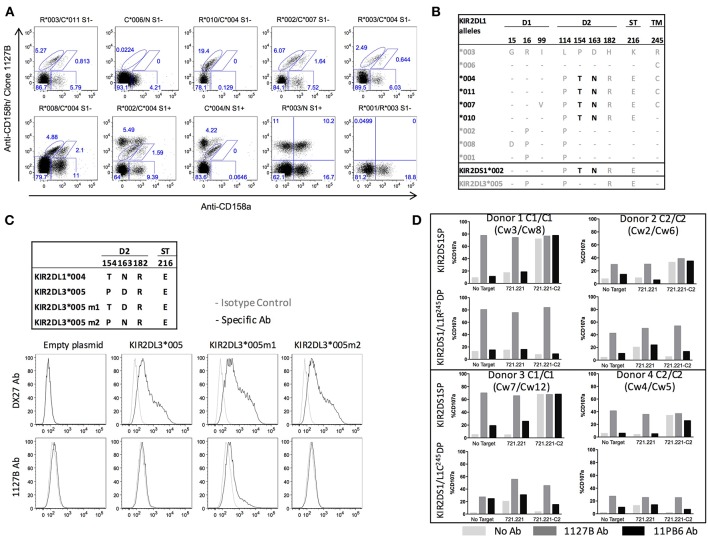
Differential binding and agonist effects of antibody 1127B to KIR2DL1 and KIR2DS1 allotypes. **(A)** Differential staining of KIR2DL1 allotypes by anti-CD158a and clone 1127B monoclonal antibodies. **(B)** Alignment of the amino acid sequences of *KIR2DL1* allotypic variants. Dashes indicate identity with the consensus KIR2DL1*003 allotype. Structural domains are indicated: Ig like domains (D1 and D2), stem domain (ST) and transmembrane domain (TM). Alleles in black encode receptor variants recognized by both anti-CD158a and clone 1127B antibody staining. Candidate amino acid residues involved in recognition by clone 1127B are indicated in black. **(C)** Binding specificities by flow cytometry of anti-CD158b1/b2/j (DX27) and clone 1127B on HEK293 cells transfected with cDNA for KIR2DL3*005 or mutation variant exhibiting amino acid substitutions in D2, as indicated. **(D)** Cytotoxic response, as measured by CD107a mobilization, in KIR2DL1^+^KIR2DS1^+^ and KIR2DS1sp cells following co-culture with 721.221 and 721.221-HLA-Cw4 target cells without additional antibody or in the presence of 1127B or 11PB6 antibody. Results are shown from four different donors, representative of ten tested.

The anti-KIR2DS1 monoclonal antibody (mAb) clone 1127B is described by the manufacturer to identify expression of KIR2DS1 and some undefined allotypes of KIR2DL1. We therefore sought to identify the KIR2DL1 allotypes that bind clone 1127B (Figure 3A). The gating of the different KIR2DL1 and KIR2DS1 subpopulations is described in Supplemental Figure 2A. We performed phenotyping on PBMC from individuals with different *KIR2DL1* alleles and found that four KIR2DL1 allotypes were recognized by clones 1127B and 143211 (^*^004, ^*^007, ^*^010, ^*^011), and five allotypes were recognized only by 143211 (^*^001, ^*^002, ^*^003, ^*^006, 008) and not by the 1127B mAb. We noted that all the KIR2DL1 allotypes recognized by the 1127B mAb are KIR2DL1 allotypes with Cys245 (C^245^), except for KIR2DL1^*^010. Furthermore, all the KIR2DL1 allotypes not recognized by the 1127B mAb are allotypes with Arg245 (R^245^), except for KIR2DL1^*^006 (Figure 3B). KIR2DL1^*^004 is the most common of the KIR2DL1-C^245^ allotypes, and compared to KIR2DL1-R^245^ allotypes, surface expression of KIR2DL1^*^004 is known to be lower (18). We also tested the 1127B mAb against KIR2DL3^*^005, previously found to be bound by the anti-KIR2DL1/S1 EB6B mAb (35), and found no specific binding by 1127B (Supplemental Figure 2B).

Aligning the protein sequences encoded by the different *KIR2DL1* alleles, with KIR2DL3^*^005 and KIR2DS1^*^002 for comparison, we identified two amino acid residues that segregate the alleles based on receptor binding by the 1127B mAb: T^154^ and N^163^ (Figure 3B). Using site-directed mutagenesis, we then altered KIR2DL3^*^005 to create a knock-in point mutation at either site to introduce the amino acid encoded in the group of alleles bound by 1127B, and tested antibody binding to HEK293 cells transfected with either mutant (Figure 3C). Neither the anti-KIR2DL2/L3/S2 mAb (clone DX27) nor the 1127B mAb bound untransfected cells, but cells transfected with either the KIR2DL3^*^005P→*T*^154^ or KIR2DL3^*^005D→*N*^163^ were bound by DX27. Only cells transfected with KIR2DL3^*^005P→*T*^154^ were bound by 1127B, indicating that it is the T^154^ amino acid that enables recognition by the 1127B mAb. As relatively uncommon alleles, KIR2DL1^*^006 is notable for being the only KIR2DL1-C^245^ allele member lacking T^154^ and N^163^, and ^*^010 is notable for being the only KIR2DL1-R^245^ allele member with T^154^ and N^163^, explaining the divergence of their antibody-binding specificities.

### *KIR2DS1* Signaling Can Override *KIR2DL1* Inhibition

That the 1127B antibody binds to only a subset of KIR2DL1 (mostly KIR2DL1-C^245^) but not to others (mostly KIR2DL1-R^245^), as well-binding to KIR2DS1, provides an opportunity to study the features of each population and the combined impacts of the same HLA-C2 signal on cells co-expressing KIR2DL1 and KIR2DS1 by flow cytometry (Supplemental Figure 3A). We selected donors positive for both *KIR2DL1 (C*^245^ *or R*^245^*)* and *KIR2DS1* from *HLA-C1/C1* or *HLA-C2/C2* individuals and challenged them with the HLA-negative target cell 721.221 or with a transfected derivative that expresses HLA-C2 (721.221-C2). We additionally included during stimulation either the 1127B antibody, or 11PB6, which universally binds KIR2DL1 and KIR2DS1 (Figure 3D).

We found that binding of the soluble 1127B mAb was able to agonize NK cells exclusively expressing KIR2DS1 (KIR2DS1sp) from *HLA-C2*-negative donors (*HLA-C1/C1*), activating them to a level equivalent to that induced by 721.221 target cells expressing the HLA-C2 ligand (Figure 3D). The agonist effect appears dose-dependent, reaching saturation at 200 ng/mL (Supplemental Figure 4). Tolerance was maintained in *HLA-C2/C2* individuals, however, and KIR2DS1sp NK cells from these donors exhibited blunted activation upon binding to either the 1127B mAb or the 721.221-C2 target cell (Figure 3D). Irrespective of their HLA-C background, cells expressing both KIR2DS1 and KIR2DL1-R^245^ were not activated upon co-culture with the 721.221-HLA-C2 target, indicating that the inhibitory signal provided by KIR2DL1-R^245^ could override target cell-induced activation signaling via KIR2DS1 upon binding to HLA-C2 in *trans*. Despite this, addition of soluble 1127B mAb, which agonizes KIR2DS1 but not KIR2DL1-R^245^, elicited activation of the double positive NK cells irrespective of donor HLA-C background, indicating that the activating signal driven by soluble 1127B is stronger than the inhibitory signal provided via KIR2DL1-R^245^ (Figure 3D). Interestingly, for KIR2DS1/L1-R^245^ double positive cells, while 1127B stimulation could overcome KIR2DL1-mediated inhibition by an HLA-C2-bearing target, the maximum antibody-induced response was still lower than that seen in the uneducated counterpart from HLA-C1/C1 cells, indicating that some measure of tolerance is maintained in KIR2DS1^+^ cells taken from an HLA-C2/C2 environment.

Using cells expressing both KIR2DS1 and KIR2DL1-C^245^, we determined that like for KIR2DS1, we could observe an agonist effect on KIR2DL1-C^245^ receptor upon binding of the 1127B antibody, due to a diminution of overall activation in the KIR2DS1^+^KIR2DL1-C^245+^ in comparison to the NK KIR2DS1sp cells (Figure 3D). This opened the opportunity to test the relative importance of inhibitory and activating signals driven by the same KIR ligand in the same NK cell. Addition of the 1127B antibody to HLA-C1/C1 NK cells positive for both KIR2DS1 and KIR2DL1-C^245^ resulted in overall activation, although the maximum activation was lower in comparison to antibody-stimulated KIR2DS1sp cells from the same individual. The agonist effects of the 1127B mAb were specific to KIR2DS1 and KIR2DL1-C^245^, as NK cells negative for KIR2DS1 and KIR2DL1 did not demonstrate activation by the 1127B mAb (Supplemental Figure 3B).

### NK Cells Educated by KIR2DL1-C^245^ Exhibit Lower “Missing Self” Reactivity Than KIR2DL1-R^245^ NK Cells

Since clone 1127B binds only a subset of KIR2DL1 allotypes, we could identify and separately study NK cells expressing different *KIR2DL1* alleles within the same individual by counterstaining with the pan-KIR2DL1 antibody 143211 (Figure 3A). This affords the opportunity to compare the function of NK cells educated in the same HLA environment by different *KIR2DL1* alleles. Importantly, all staining for KIR2DL1, including with the 1127B antibody, was completed after stimulation; therefore, the antibodies could not impact the reactivity of NK cells.

**Figure 4:**
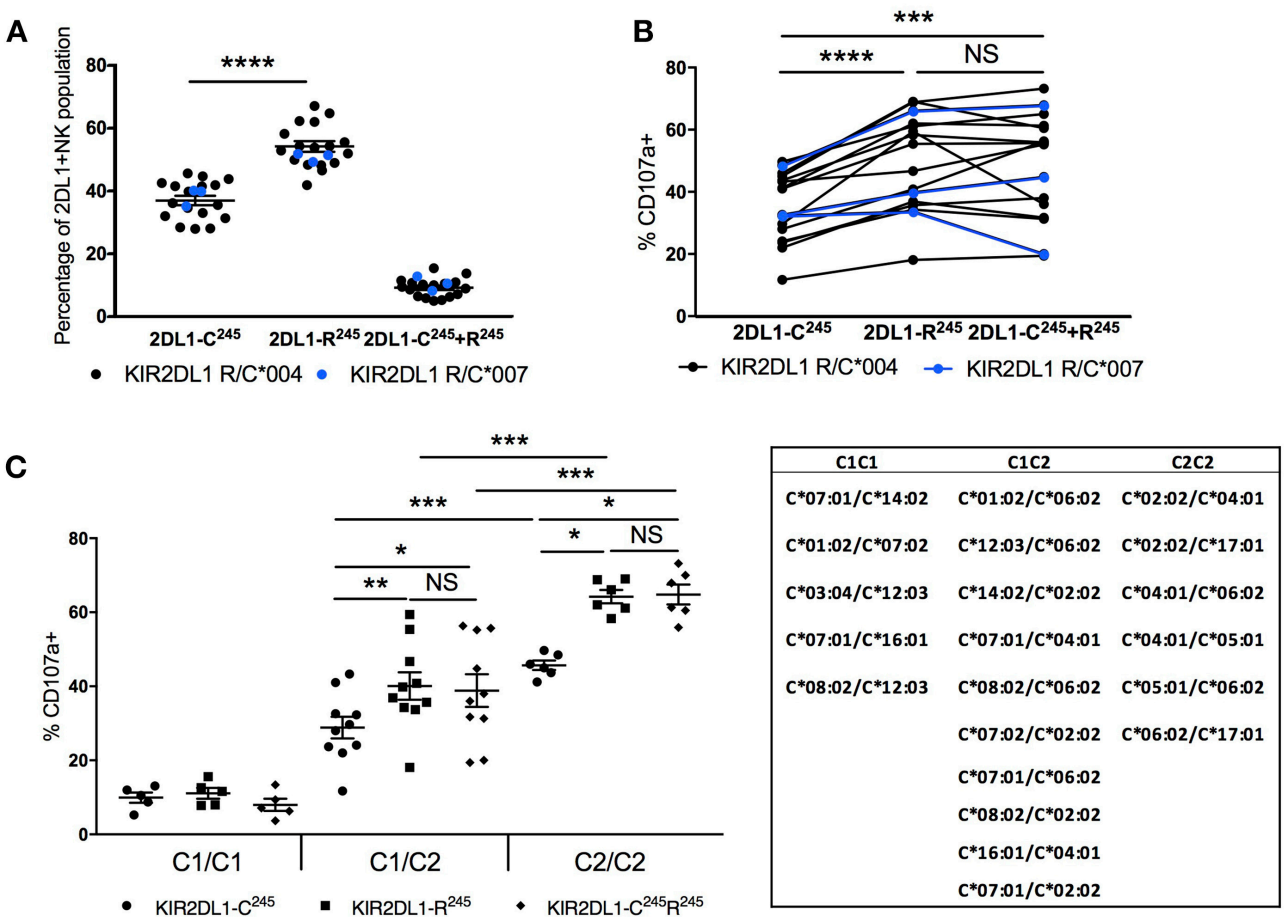
Impact of *KIR2DL1* allelic diversity and *HLA-C2* copy number on NK education. Cytotoxic response, measured by CD107a mobilization following 5-h co-culture with K562 target cells, was evaluated on NK cells single positive for KIR2DL1 and negative for other inhibitory KIR and NKG2A. **(A)** Frequencies of NK populations with single positive or double positive for KIR2DL1-C^245^ and KIR2DL1-R^245^ allotypes among individuals with both. KIR2DL1-C^245^ allotypes *004 and *007 display similar expression frequencies. **(B)** Hierarchical differences in cytotoxic response among NK populations single positive for KIR2DL1-C^245^ or KIR2DL1-R^245^ allotypes or double positive for both. **(C)** Impact of *HLA-C2* copy number on the education of NK cells expressing KIR2DL1-R^245^ and KIR2DL1-C^245^ allotypes. Donor HLA-C allotypes are listed. Paired and unpaired Mann-Whitney tests were used. Symbols represent individual samples (mean ± SEM). *p < 0.05, **p < 0.01, ***p < 0.001, and ****p < 0.0001.

We first analyzed the frequency of the different subpopulations of NK cells expressing KIR2DL1 in individuals co-expressing either KIR2DL1^*^004 or ^*^007 (both KIR2DL1-C^245^ allotypes) and a KIR2DL1-R^245^ allotype (Figure 4A). The percentage of NK cells that express KIR2DL1^*^004 or ^*^007 was lower in comparison to the percentage of cells expressing KIR2DL1-R^245^ (*p* < 0.0001), confirming our earlier results observed in individuals homozygous and hemizygous for *KIR2DL1* (Figure 1A). Co-expression of two KIR2DL1 allotypes was uncommon regardless of KIR2DL1-C^245^ or -R^245^ categorization and was observed on an average of 9.11 ± 0.64% of all KIR2DL1^+^ NK cells.

We compared the functional capacity of NK cells expressing different KIR2DL1 allotypes by measuring degranulation response (CD107a) in response to challenge with the HLA-negative target cell line K562 for 5 h. Using donors that carried both *KIR2DL1-R*^245^ and –*C*^245^ alleles, we could examine within the same donor, functional differences between NK populations expressing each KIR2DL1 receptor allotype (Figure 4B). We analyzed NK cells singly expressing KIR2DL1, excluding cells co-expressing other inhibitory receptors that could contribute to NK education. We found that NK cells expressing KIR2DL1-C^245^ (KIR2DL1^*^004 or ^*^007) allotypes displayed a lower frequency of degranulation in comparison to the NK cells expressing KIR2DL1-R^245^ allotypes within the same donor (*p* < 0.0001). NK cells co-expressing both alleles had a similar pattern of degranulation to cells expressing only KIR2DL1-R^245^ allotypes, suggesting a dominant program of education by KIR2DL1-R^245^.

### *HLA-C2* Copy Number Calibrates the Education of *KIR2DL1*-Expressing NK Cells

We next analyzed the same functional data, grouping donors based on genomic *HLA-C2* copy number (Figure 4C). Compared with donors exhibiting two copies of *HLA-C2* alleles, KIR2DL1^+^ NK cells from donors having only one copy of *HLA-C2* exhibited lower degranulation in response to K562 target cells. This differential function was observed for the NK cells expressing KIR2DL1-C^245^ (*p* < 0.001) or KIR2DL1-R^245^ (*p* < 0.001) or both (*p* < 0.001) and suggests that the education of KIR2DL1^+^ NK cells is instructed by the quantity of available ligand, similar to the partnership of KIR3DL1 and HLA-Bw4 (11).

### KIR2DL1-C^245^ and KIR2DL1-R^245^ Receptors Are Similarly Inhibited by HLA-C2 and Not Internalized in the Presence of Their Ligand

**Figure 5:**
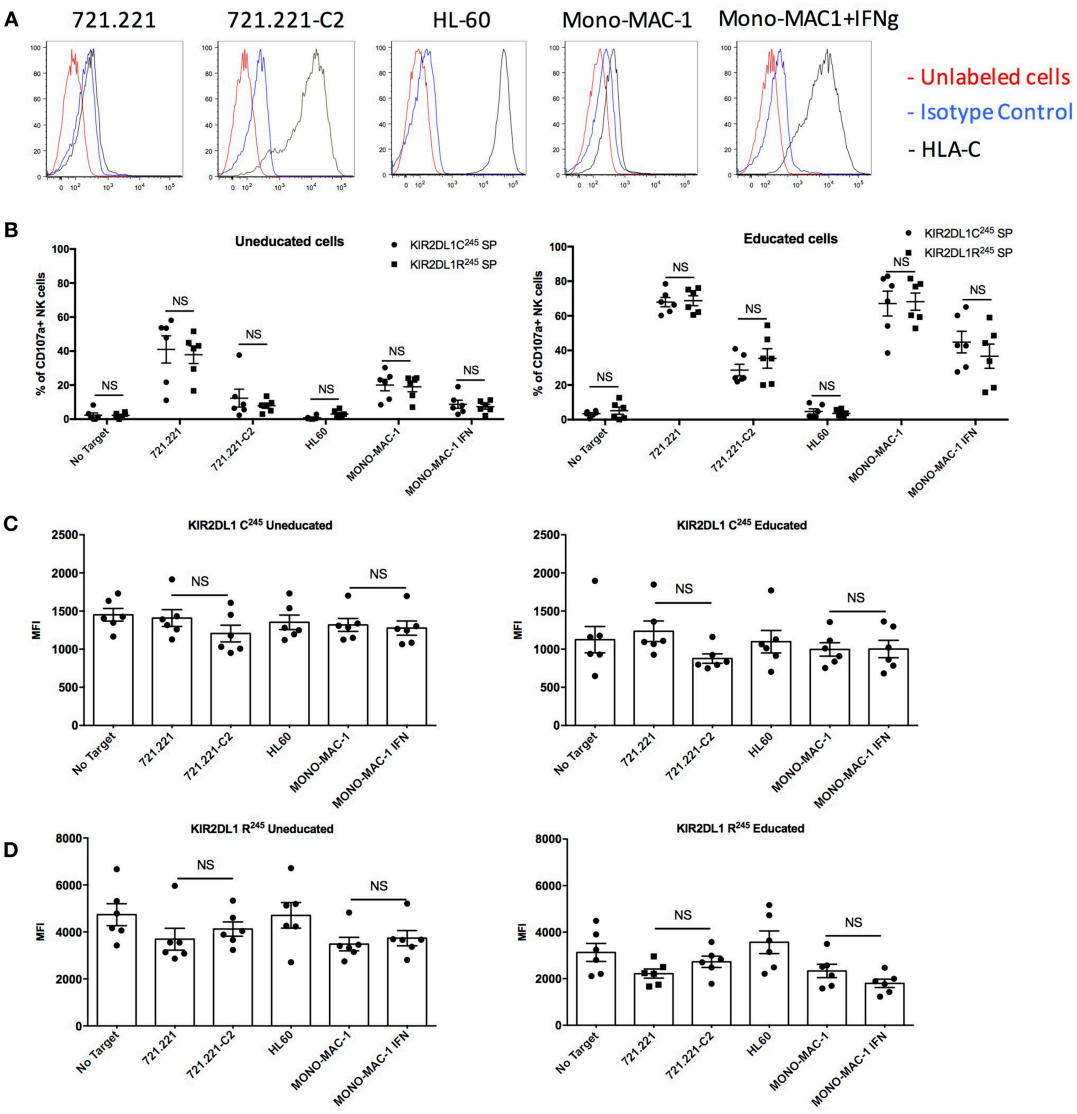
Inhibitability of the KIR2DL1 allotypes following 24 h of co-culture with HLA-C2^+^ target cells. **(A)** Cell surface expression of HLA-C on the HLA-class I negative 721.221 target cell, 721.221 transfected with HLA-Cw2 (721.221-C2), and the HLA-C2-bearing AML cell lines HL-60 and Mono-MAC-1 with or without treatment with IFN-γ. **(B)** Cytotoxic response and inhibition among educated and uneducated NK populations expressing different KIR2DL1 alleles to 721.221 and the HLA-C2 ligand-expressing target cells, as indicated. **(C)** Surface MFI of KIR2DL1-C^245^ receptors following incubation with ligand-expressing target cell. **(D)** Surface MFI of KIR2DL1-R^245^ receptors following incubation with ligand-expressing target cells. Unpaired and paired Mann-Whitney tests were used. Symbols represent individual samples (mean ± SEM).

Using cells transfected to express either the *KIR2DL1-C*^245^ *or -R*^245^ alleles, a previous study reported that KIR2DL1 isoforms conveyed different sensitivities to inhibition potentially due to differences in receptor internalization after 24 h incubation with HLA class I expressing targets (16). To study the relevance of this finding in primary human NK cells, we performed a similar 24 h target cell co-incubation, comparing degranulation of KIR2DL1^+^ NK cells from 12 individuals co-expressing the KIR2DL1-C^245^ allele KIR2DL1^*^004 and a KIR2DL1-R^245^ allele, including individuals with or without the cognate HLA-C2 ligand. Target cells included the 721.221 cell line transfected with an *HLA-C2* allele and the HLA-C2^+^ acute myelogenous leukemia cell lines HL-60 (HLA-C2/C2) and MONO-MAC-1 (HLA-C1/C2). HLA expression is high without additional stimulation of the HL-60 cell line, and exposure to IFN-γ induced upregulation of HLA-C on the MONO-MAC-1 leukemia cell lines (Figure 5A). As expected, and reflecting their lack of education, KIR2DL1^+^ NK cells from *HLA-C1/C1* donors were hyporesponsive compared to those from *HLA-C2*^+^ donors against 721.221 target cells and MONO-MAC-1 cells unstimulated with IFN-γ. In contrast, KIR2DL1^+^ NK cells from *HLA-C2*^+^ donors were responsive against 721.221 and unstimulated MONO-MAC-1 cells, and inhibited by HLA-C2^+^ target cells, including the 721.221-C2 transfectant, HL-60 cells and IFN-γ-stimulated MONO-MAC-1 cells to differing degrees (Figure 5B). Using the 24 h co-incubation condition and in contrast to the previous study (16), we observed no difference in degree of inhibition of NK cells expressing the KIR2DL1-C^245^ allele KIR2DL1^*^004 compared to those expressing a KIR2DL1-R^245^ allele (Figure 5B). Differences in degranulation between NK cells expressing the different KIR2DL1 subtypes also decreased after 24 h co-incubation, in contrast to what we observe after the more conventional 5 h co-culture condition, demonstrating that less educated NK cells can achieve the same degree of activation as more educated cells given longer stimulation (Supplemental Figures 5A,B).

We measured the cell surface expression of the KIR2DL1-C^245^ KIR2DL1^*^004 allele and of the KIR2DL1-R^245^ alleles after 24 h of co-culture in the presence or absence of HLA-C2-expressing target cells. The results showed equivalent cell surface expression with or without target cells expressing the *HLA-C2* ligand, for cells expressing KIR2DL1-C^245^ KIR2DL1^*^004 (Figure 5C and Supplemental Figure 5C) or a KIR2DL1-R^245^ allele (Figure 5D and Supplemental Figure 5C). These results contrast with a previous study using an NK cell line transfected with each of the two KIR2DL1 isoforms (16), and suggest that both are, in fact, stably present on the cell surface on primary NK cells.

### KIR2DS1**-Mediated Education Is Titrated by *HLA-C2* Copy Number and by NKG2A

**Figure 6:**
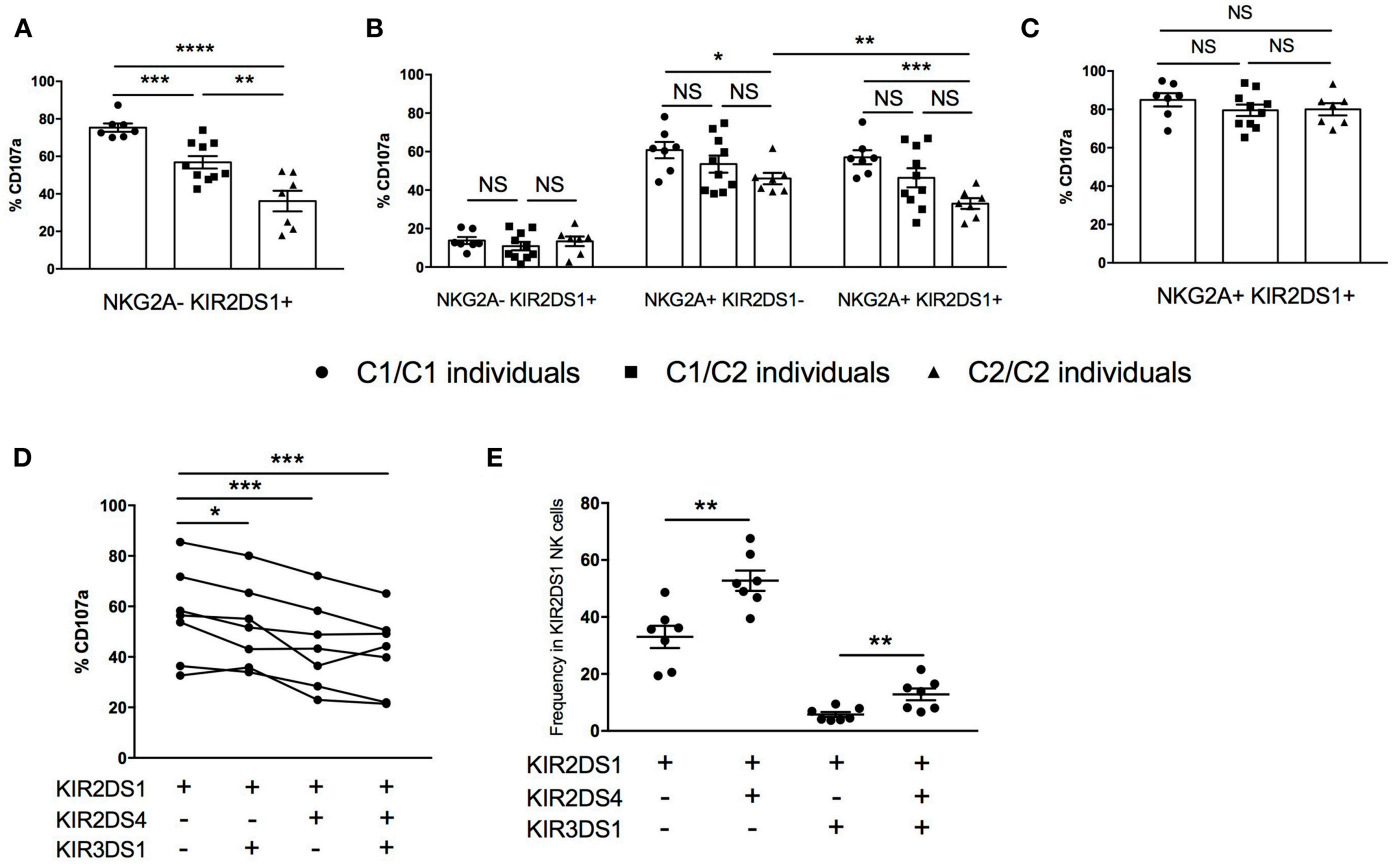
KIR ligand titration of KIR2DS1^+^ NK tolerance, integration of NK education via NKG2A, and co-expression of other activating KIR. **(A)** Cytotoxic response among KIR2DS1sp cells following activation by 721.221-Cw4 cells, segregated by autologous HLA-C KIR ligand genotype. **(B)** Response capacity of KIR2DS1^+^ and/or NKG2A^+^ NK cells to 721.221 target cells, segregated by autologous HLA-C KIR ligand genotype. **(C)** Cytotoxic response of KIR2DS1^+^NKG2A^+^ to 721.221-Cw4, segregated by autologous HLA-C KIR ligand genotype. **(D)** Response capacity of KIR2DS1^+^ cells co-expressing KIR2DS4 and/or KIR3DS1 to 721.221 target cells **(E)** Relative frequencies of KIR2DS1 NK cells co-expressing KIR2DS4 and/or KIR3DS1. Unpaired and paired Mann-Whitney test were used. Symbols represent individual samples (mean ± SEM). *p < 0.05, **p < 0.01, ***p < 0.001, and ****p < 0.0001.

The activating KIR2DS1 receptor also impacts the education of NK cells (4, 5). In contrast to inhibitory KIR2DL1, which programs higher education and responsiveness in the presence of autologous ligand HLA-C2, KIR2DS1 renders the cell more tolerant when it is expressed in the presence of the same ligand. We compared the reactivity of KIR2DS1^+^ NK cells against 721.221 target cells transfected with the activating ligand HLA-C2, stratifying our analysis based on the donor's *HLA-C2* copy number (Figure 6A and Supplemental Figure 6A). In *HLA-C2/C2* individuals, NK cells exclusively expressing KIR2DS1 were the least responsive cells, consistent with tolerization of this population (4, 5). In contrast, the most responsive KIR2DS1^+^ NK cells were from donors lacking *HLA-C2* (*HLA-C1/C1*), while *HLA-C1/C2* individuals exhibited intermediate reactivity compared to both groups, demonstrating that tolerance or “negative education” is titratable by environmental HLA.

Studies examining the education of NK cells based on expression of a single educating receptor are vital to our understanding of the contribution of individual receptor-ligand pairs to NK education, but the majority of NK cells in the human repertoire co-express multiple KIR and/or NKG2A (17, 36). To better understand the impact of KIR2DS1 and HLA-C2 on NK cell function in a more physiologically relevant context, we analyzed NK cells co-expressing the educating activating receptor KIR2DS1 and inhibitory NKG2A. Against the HLA-negative 721.221 target cell line, NKG2A^+^ single-positive NK cells from *HLA-C2/C2* donors trended toward less degranulation than those collected from *HLA-C1/C2* or *HLA-C1/C1* donors (Figure 6B). Among the same *HLA-C2/C2* individuals, NK cells co-expressing NKG2A, and KIR2DS1 were even less responsive than NKG2A single positive cells, demonstrating that the tolerization of KIR2DS1 by HLA-C2/C2 can diminish NK cell education by NKG2A (*p* < 0.01) (4).

When the same cells were used to compare reactivity against 721.221-C2 cells, we were surprised to find that where KIR2DS1^+^ NK cells were previously demonstrated to be tolerized (e.g., in an *HLA-C2/C2* individual), we now found equal and strong reactivity among all KIR2DS1^+^NKG2A^+^ NK cells, irrespective of the donor HLA-C background, suggesting that education through NKG2A overrides the tolerization driven by KIR2DS1 in *HLA-C2/C2* donors (Figure 6C and Supplemental Figure 6B).

Activating KIR molecules other than KIR2DS1 are not known to contribute to NK education, either in a potentiating or tolerizing fashion, but they have not previously been examined systematically. Using KIR2DS1-mediated NK cytotoxic response to HLA-C2 on the target cell for comparison, we examined how, within the same individuals, co-expression with KIR2DS1 of the activating receptors KIR2DS4 or KIR3DS1 on the NK cell impacts responsiveness to the same HLA-C2^+^ target. Interestingly, co-expression of either KIR2DS4 or KIR3DS1 reduced the likelihood of a KIR2DS1-expressing NK cell to respond, as measured by CD107a staining (Figure 6D). Importantly, there was no reduction in MFI of KIR2DS1 in the setting of a co-expressed activating receptor (data not shown). Notably, contrary to the product rule for co-expression of additional KIR (37), KIR2DS1^+^ NK cells were unexpectedly more likely to co-express KIR2DS4 than not (Figure 6E). This was not the case, however, for KIR3DS1, which was rarely co-expressed with KIR2DS1. In comparison, there was a higher frequency of cells expressing all three activating KIR, suggesting the preferential co-expression of KIR2DS4^+^ if KIR2DS1 is expressed, even with KIR3DS1. Therefore, in individuals with *KIR2DS1* and *KIR2DS4* in their genotype, KIR2DS1^+^ NK cells are more likely to co-express KIR2DS4, potentially resulting in dampened repertoire response to HLA-C2 activation.

### KIR2DL1^+^ NKG2C*^+^* NK Expansion in HCMV^+^ Individuals can be Mono- or Bi-allelic

Previous studies have demonstrated expansion of NKG2C-expressing NK cells among some individuals previously exposed to HCMV; in particular, these “adaptive NK” cells most often co-express inhibitory KIR2DL molecules educated by “self” HLA-C molecules (17). Since we found that KIR2DL1-C^245^ and KIR2DL1-R^245^ allotypes differently educate NK cells for missing self-reactivity, we explored whether that the magnitude of NK education also impacts selective expansion of NKG2C^+^ NK cells.

**Figure 7:**
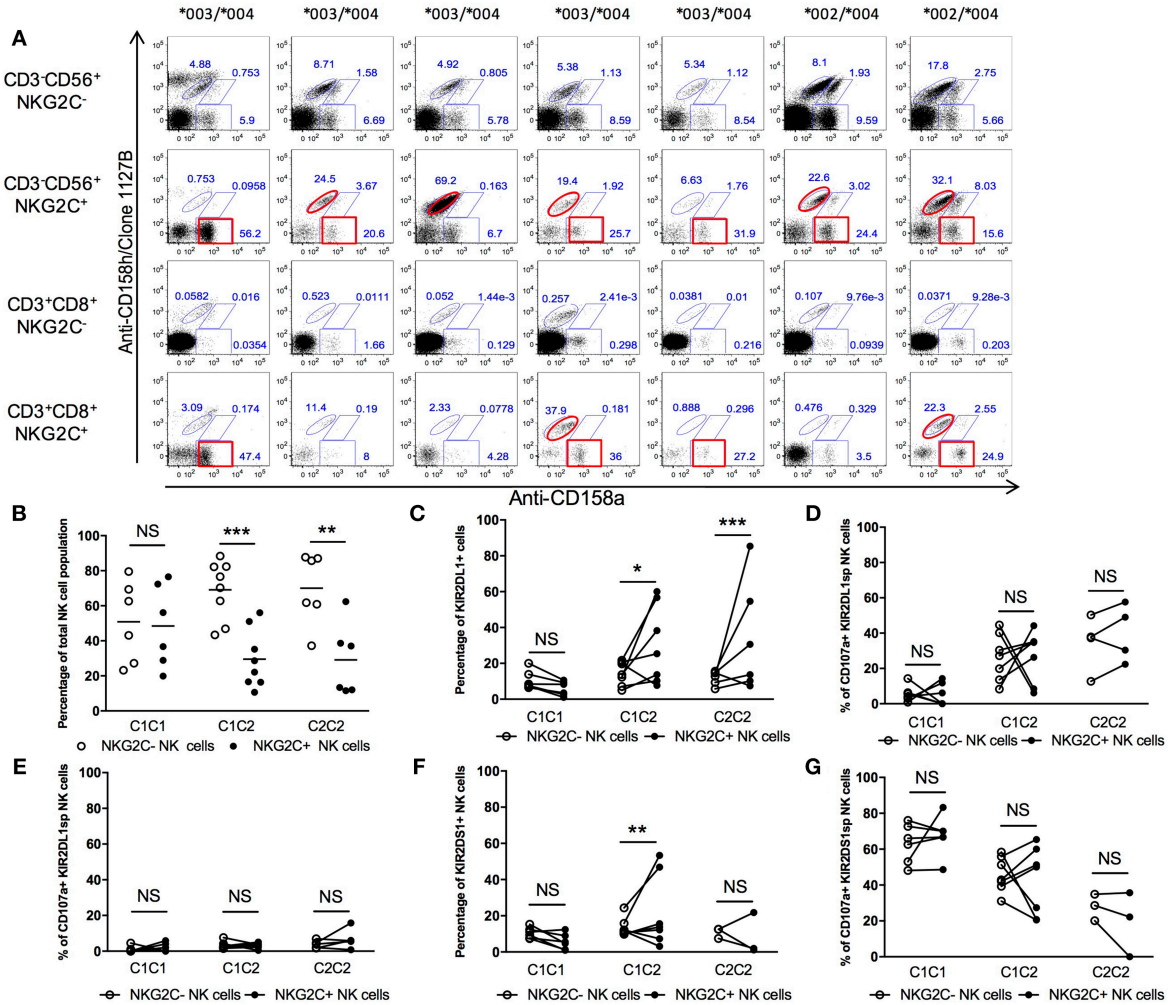
KIR2DL1-S1 expression and function in NKG2C^+^ compartments. **(A)** Differential KIR2DL1 allele frequencies in NKG2C^+^ NK cells. **(B)** Frequency of NKG2C^−^ and NKG2C^+^ NK compartments in HCMV^+^ healthy donors. **(C)** Frequency of KIR2DL1^+^ cells within the NKG2C^−^ and NKG2C^+^ NK cell compartments. **(D)** Responsiveness of KIR2DL1SP NK cells in NKG2C^−^ and NKG2C^+^ compartment, against 721.221. **(E)** Inhibition of KIR2DL1sp cells in NKG2C^−^ and NKG2C^+^ compartment, with 721.221-Cw4. **(F)** Frequency of KIR2DS1^+^ cells in NKG2C^−^ and NKG2C^+^ NK populations. **(G)** Cytotoxic responsiveness of KIR2DS1sp with 721.221-Cw4 cells. Unpaired Mann-Whitney test was used. Symbols represent individual samples (mean ± SEM). *p < 0.05, **p < 0.01, and ***p < 0.001.

We analyzed seven different individuals with an NKG2C+ population and having at least one copy of *HLA-C2* and co-inheriting both KIR2DL1-C^245^ and KIR2DL1-R^245^ allotypes to determine whether higher education and stronger “missing self” reactivity was preferentially associated with expansion of NKG2C after HCMV infection (Figure 7A). Within seven samples, we found four with a co-dominant KIR2DL1 allotype NK expansion, two with a preferential KIR2DL1-R^245^ allotype expansion and one with a preferential KIR2DL1-C^245^ expansion (Figure 7A). The same observation was made in NKG2C^+^ CD8^+^ T cells, but the particular isoform expansion was not necessarily the same in T and NK cells from the same donor (Figure 7A). In HCMV-seropositive individuals, despite the preferential expression of self-specific educating KIR in the adaptive NK cell compartment, there is no apparent preferential expression of the KIR2DL1 allele conferring higher education.

### NKG2C Expression Does Not Modulate the Function of KIR2DL1^+^ or KIR2DS1^+^ NK Cells in a Predictive Way

Recent studies characterized phenotypic and functional differences between adaptive and non-adaptive NK cells (17, 34, 38, 39). We examined if the function of KIR2DL1^+^ and KIR2DS1^+^ cells differed between the adaptive NK population compared to the non-adaptive NK population.

Cells from 19 HCMV-seropositive individuals, all of whom displayed an adaptive NKG2C^+^ NK population, were co-cultured with 721.221 or with the 721.221-C2 transfectant (Figure 7B). It should be noted that while in this small cohort, HLA-C2^+^ donors displayed an overall lower NKG2C^+^ NK cell population in comparison to HLA-C1 donors, this was not confirmed in a larger cohort (data not shown). We analyzed the frequency of KIR2DL1^+^ NK cells among the NKG2C^+^ NK population and, consistent with their educated phenotype, found KIR2DL1^+^ NKG2C^+^ NK cells only in individuals having at least one HLA-C2 ligand for KIR2DL1 (Figure 7C). We found that KIR2DL1sp cells had on average the same degranulation against 721.221 in comparison to those co-expressing NKG2C^+^ but with important variations within individuals (Figure 7D). We did not observe any difference in inhibition by 721.221-C2 between adaptive and non-adaptive KIR2DL1^+^ NK cells (Figure 7E). We also only observed the KIR2DS1^+^NKG2C^+^ NK cell population in HLA-C2^+^ donors (Figure 7F), the first evidence that the presence of ligand can influence expansion of activating KIR^+^ cells within the adaptive NK repertoire of HCMV-seropositive individuals and suggesting possible auto-reactivity of KIR2DS1+ NK cells partially driven by HLA-C2. In co-culture with 721.221-C2, KIR2DS1^+^NKG2C^+^ NK cells did not behave differently in average from KIR2DS1sp cells but with important variations within individuals (Figure 7G). We conclude that KIR2DS1^+^ NK cell expansion in HCMV-seropositive individuals is only observed in HLA-C2 individuals, and NKG2C does not impact NK cell function in a predictive way.

## Discussion

*KIR2DL1* and *KIR2DS1* allelic variation and copy number and the availability of their ligand, HLA-C2, combine to impact the phenotype and function of primary human NK cells. Compared with KIR2DL1-C^245^, KIR2DL1-R^245^ allotypes are expressed at higher cell surface densities and convey greater missing self-reactivity when educated by HLA-C2 group ligands, consistent with previous findings (14). Despite this, the KIR2DL1 isoforms similarly signal inhibition of NK cells, including those co-expressing the activating KIR2DS1 receptor. While KIR2DL1 is expressed on the expanded NKG2C^+^ population in HCMV-seropositive, *HLA-C2*^+^ donors, neither KIR2DL1-C^245^ nor KIR2DL1-R^245^ isoform exhibits dominance among the NKG2C^+^ adaptive NK population. Together, our findings suggest that KIR2DL1 isoforms co-evolved with HLA-C2 to enable differential detection of and response to target cells missing self HLA, while maintaining a dominant sensitivity for inhibition to avoid autoimmunity.

Using a combination of commercially available monoclonal antibodies including the 1127B clone, which we show to be specific for T^154^ present in KIR2DL1-C^245^ allotypes (except for ^*^006), but not KIR2DL1-R^245^ allotypes (except for ^*^010), we compared function between NK cells expressing KIR2DL1-R^245^ and cells expressing KIR2DL1-C^245^ in heterozygous donors. Primary, unmodified NK cells, educated in a shared HLA-C2^+^ environment, offered the opportunity to directly compare NK cell education imparted by the different allotypes. We found that *KIR2DL1* alleles are expressed at predictable cell surface densities and on predictable frequencies of NK cells and that KIR2DL1 is expressed in a primarily monoallelic fashion per cell. Co-expression of both KIR2DL1 isoforms was found on just ~9% of the KIR2DL1^+^ NK cell population, consistent with the product rule for receptor expression and co-expression (37). Moreover, our results confirm a linear correlation between *KIR2DL1* and *KIR2DS1* allele copy number and the frequency of NK cells that express these receptors (30), where specific *KIR2DL1* allele subtypes are associated with low (*KIR2DL1-C*^245^) or high (*KIR2DL1-R*^245^) cell surface expression and frequency within the NK repertoire (18, 40). Therefore, allele variation is a major determinant of the likelihood of KIR2DL1 expression on any given NK cell and the extent to which KIR2DL1 is expressed on its cell surface.

We observed an agonistic effect of soluble 1127B mAb on KIR2DL1 and KIR2DS1 receptors. This result is unique to the 1127B mAb and was not recapitulated by 11PB6, which instead binds and blocks KIR2DL1 and KIR2DS1. Previous reports have demonstrated the importance of the D1 domain for epitope-specific recognition of the HLA-C ligand by KIR2D receptors (9, 41). Here we show threonine at position 154 in D2 is critical for the binding of 1127B antibody to the D2 domain. 1127B binding of KIR2DS1 is sufficient to mimic the binding of HLA-C2, maintaining the hyporesponsiveness induced by the expression of HLA-C2 by the donor. This finding is in apparent contrast to a previous study, which showed that KIR2DS1^+^ cells could be induced for responsiveness by 11PB6-mediated crosslinking, with no hyporesponsiveness among *HLA-C2/C2* donors compared to *HLA-C1/C1* donors (4). Using the agonist effect of the 11217B mAb, we observed that activation through KIR2DS1 can override KIR2DL1 on NK cells co-expressing KIR2DS1 and KIR2DL1-R^245^ upon challenge with a HLA-C2^+^ target, demonstrating how strong activating signaling can overcome inhibitory signaling. The 1127B mAb appears to have some agonistic inhibitory properties upon binding KIR2DL1-C245, as we observe an attenuated response in HLA-C1/C1 individuals to 1127B stimulation of KIR2DS1^+^KIR2DL1-C^245+^ NK cells in comparison to KIR2DS1sp from the same individual. Responses to antibody stimulation in KIR2DS1^+^KIR2DL1-C^245+^ NK cells from HLA-C2/C2 individuals were even more blunted. Together, these studies indicate that KIR2DS1 and KIR2DL1 signaling can proceed independently and suggest that the strength of incoming signals determines the reactivity of NK cells. Strong activating signals can still be generated despite KIR2DS1^+^ NK cell tolerization in HLA-C2/C2 hosts, indicating that tolerization does not impact the innate response capacity, but that the degree of response is driven by the nature of the stimulus. Simultaneous engagement of inhibitory and activating receptors by ligand on a target cell leads to inhibition overruling activation; whereas simultaneous agonist antibody engagement of inhibitory and activating receptors leads to activation overruling inhibition.

We find that *KIR2DL1* and *KIR2DS1* alleles are naturally expressed at different cell surface densities, and by comparing NK cells educated in different HLA-C environments and by the same *KIR2DL1* isoforms, we can conclude that *HLA-C2* diminishes the brightness of KIR2DL1 but not KIR2DS1 staining on NK cell surfaces in a dose-dependent manner. A diminution of inhibitory receptor staining has been observed in mice, where education of NK cells is associated with lower MFI of the inhibitory Ly49 receptors that engage MHC class I molecules (42, 43) Molecular studies in mice indicate that this phenotypic change results from *cis* interactions between the cognate receptor-ligand partnerships that interfere with antibody binding (44–46). Functionally, these interactions interfere with engagement of Ly49 by inhibitory ligands available from neighboring cells in *trans*, increasing the threshold for NK cell inhibition (47, 48). *Cis* associations between KIR and HLA have not directly been demonstrated, and the KIR proteins are non-orthologous to Ly49 (49). However, KIR3DL1^*^004, a stable KIR3DL1 allotype that is retained intracellularly, appears to educate NK cells in the presence of its cognate HLA-Bw4 ligand (10), and the KIR ligand in *cis* has a profound influence on long-term functionality of NK cells (50). Furthermore, HLA-C expression is modulated specifically in NK cells that express its cognate KIR receptor (51). Therefore, KIR-HLA interactions may occur intracellularly and could impact the availability of KIR proteins for antibody staining and for surface interaction with ligands in *trans*. HLA-C2 polymorphism could differentially impact KIR2DL1 expression, as HLA-C2 alleles have different densities of expression (52) and different affinities for KIR2DL1 alleles (9).

In individuals exhibiting genes encoding KIR2DL1 and its educating HLA-C2 ligand, the extent of NK education, measured as “missing self” responsiveness, correlates with the cell surface density of the inhibitory receptor: KIR2DL1^+^ NK cells exhibit greater MFI and missing self-responsiveness when they are educated by the high density KIR2DL1-R^245^ isoforms compared with the low density KIR2DL1-C^245^ isoforms. We have similarly reported that KIR3DL1 receptors expressed at the highest cell surface densities exhibit the highest response capacity, especially when coupled with high-density isoforms of their cognate HLA-Bw4 ligand (11). These findings in human NK education correspond to observations in mice, in which the density of Ly49 receptors educated by “self” MHC class I also corresponds with increasing education (53, 54).

We found that the copy number of *HLA-C2* increases NK cell education in a dose-dependent manner. Currently, there are no antibodies available to distinguish subtypes of HLA-C, or to distinguish the HLA-C2 and HLA-C1 group members which do or do not, respectively, ligate KIR2DL1. Nevertheless, this finding suggests that, as for HLA-Bw4 copy number and KIR3DL1^+^ NK cells (11), response capacity in KIR2DL1^+^ cells is tuned to the availability of cognate ligand at steady state. We extend this finding to the activating receptor, KIR2DS1, where the reactivity of KIR2DS1-expressing NK cells is negatively attenuated in a fashion commensurate with the gene “dose” of HLA-C2 ligand. These findings are in contrast to an earlier report, in which clonally-expanded KIR2DS1sp NK cells were only found to be tolerized in donors with two copies of *HLA-C2* (5); using primary cells, we observe that while the most profound diminution of response is found among *HLA-C*2 homozygotes, we can identify an intermediate educational level conferred by a single copy of HLA-C2 ligand, consistent with a ligand-associated titration of effector response. Further, we find that this “tolerization” extends to cells co-expressing other self-sensitive inhibitory receptors, as NKG2A^+^KIR2DS1^+^ cells from *HLA-C2/C2* individuals exhibit a reactivity lower than NKG2Asp cells or KIR2DS1^+^ cells from *HLA-C1/C1* individuals.

We observed a lower education of NKG2Asp^+^ NK cells from *HLA-C2/C2* donors in comparison to those from *HLA-C1/C2* or *HLA-C1/C1* donors. The difference in education is presumably due to dimorphism at position−21 in the leader sequence of HLA-B, which has been found to be associated with education of NKG2A-bearing NK cells (55). A threonine residue at−21 (-21T) does not deliver functional peptides to HLA-E, leading to lower education for NKG2A in individuals bearing HLA haplotypes with−21T HLA-B molecules, which, by positive linkage disequilibrium, are enriched for HLA-C2. The pairing of HLA-C2 and poor NKG2A education through−21T likely accounts for the lower education of NKG2Asp cells from *HLA-C2C2* donors.

While we observed an impact of *HLA-C2* copy number and *KIR2DL1* allele subtype on primary NK effector response capacity, we did not observe a similar difference in sensitivity to inhibition by the HLA-C2 targets allotypes tested in our study. These findings lead us to conclude that in contrast to previously published studies using transfected cells (16), primary NK cells exhibiting KIR2DL1-C^245^ or KIR2DL1-R^245^ are similarly sensitive to inhibition. Further investigation is necessary to determine if primary NK cells expressing KIR2DL1-C^245^ or KIR2DL1-R^245^ exhibit different capacities to kill tumor cells *in vivo*.

Until recently, it has been challenging to study the function of highly homologous activating and inhibitory KIR receptors and to distinguish between KIR allotypes on primary NK cells due to the lack of reagents and methodologies. In this study, we propose the combination of two antibodies (1127B and 143211 mAb) to separate KIR2DS1 and the two most common KIR2DL1 allele subtypes at the same time. Other studies have proposed alternative antibody strategies to separate KIR2DS1 and KIR2DL1 or KIR2DL1 alleles (4, 14); and still other combinations of commercially available antibodies can separate the KIR2DL2/2DS2 receptor pair (56).

The ability to dissect KIR2DL1 subtypes by flow cytometry is an important facet of this investigation, because it permits assessment of primary NK cells expressing different KIR2DL1 allotypes but educated in the same HLA-C environment. In this way, we can consider whether the expansion of self-specific KIR^+^ cells in response to HCMV infection that has been previously described (17) is related to the magnitude of reactivity of NK cells. Our results demonstrate instead that there is not preferential expansion of the more educated, more responsive KIR2DL1-R^245+^ population over the less responsive KIR2DL1-C^245+^ population. Interestingly, like KIR2DL1, KIR2DS1 is found on NKG2C^+^ cells only from individuals expressing HLA-C2.

The expansion of NKG2C^+^ NK subsets in response to HCMV infection is well-documented (17, 57), but how this population interacts with infected cells and impacts viral control is not well-understood. That HMCV-infected cells exhibit self HLA class I molecules and NKG2C^+^ NK cells exhibit cognate inhibitory KIR suggests that HMCV-infected cells could drive inhibition of educated NK cells. In our present study, we did not observe preference of any KIR2DL1 allele for expansion on NKG2C^+^ cells in donors genotyped for both KIR2DL1-R^245^ and KIR2DL1-C^245^. Whether subtle differences in the capacity for inhibition, which could be mediated by the particular alleles of HLA-C in each person, or input from other co-expressed receptors, and the impact of *KIR2DL1* in combination with *NKG2C* on autologous cells infected with HCMV, remain to be further investigated.

Control of cancer, infection, autoimmunity, and reproduction have all been linked to *KIR-HLA* partnerships (3), and we have recently demonstrated that allele subtype variation for *KIR3DL1* and *HLA-Bw4* can have similar impacts (10, 12). That *KIR2DL1, KIR2DS1*, and *HLA-C2* have co-evolved, diversified and enable variable NK cell function is intriguing and may similarly reflect distinctions in the capabilities of NK cells for maintenance of human health.

## Ethics Statement

Peripheral blood samples were collected from healthy human donors following approval from the Memorial Sloan Kettering Cancer Center (MSKCC) Institutional Review Board, and donors provided informed, written consent. The MSKCC IRB waived the need for additional research consent for anonymous NYBC samples.

## Author Contributions

J-BL designed the study. J-BL and JF performed the experiments. J-BL and JB created the bank samples. J-BL, JB, and KH wrote the manuscript. All authors discussed the results and contributed to the final manuscript. KH supervised the project.

### Conflict of Interest Statement

The authors declare that the research was conducted in the absence of any commercial or financial relationships that could be construed as a potential conflict of interest.
