# Supplementary material for: Novel Approach to Cell Surface Discrimination Between KIR2DL1 Subtypes and KIR2DS1 Identifies Hierarchies in NK Repertoire, Education, and Tolerance
Source: Front Immunol. 2019 Apr 5;10:734. doi: 10.3389/fimmu.2019.00734 (PMC6460669; doi:10.3389/fimmu.2019.00734)

Novel approach to cell surface discrimination between KIR2DL1 subtypes and KIR2DS1 identifies hierarchies in NK repertoire, education and tolerance

Jean-Benoît Le Luduec, Jeanette E. Boudreau, Julian C. Freiberg, Katharine C. Hsu

Supplemental Figures

Supplemental Figure 1: NK cell surface expression of KIR2DL1*002. Cells were stained with anti-KIR2DS1 (1127B) in addition to anti-KIR2DL1/S1 (EB6B) to gate out the KIR2DS1^{+} and KIR2DL1^{+}/S1^{+} NK cells. The figure represents the MFI of NK cells bearing KIR2DL1*002 alleles, as stained by EB6B. Unpaired Mann-Whitney test was used. Symbols represent individual samples (mean ± SEM). ** *p<0.01*.

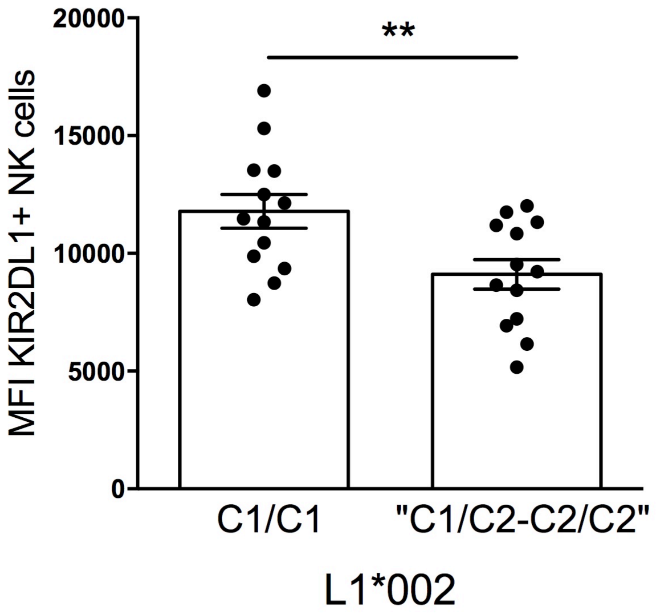

Supplemental Figure 2: Binding of 1127B mAb. (**A**) Separation of KIR2DL1 and KIR2DL1 subpopulations with 1127B and 143211 mAb combination. (**B**) Differential binding of EB6B and 1127B mAb on cells expressing KIR2DS1 and KIR2DL3*005.

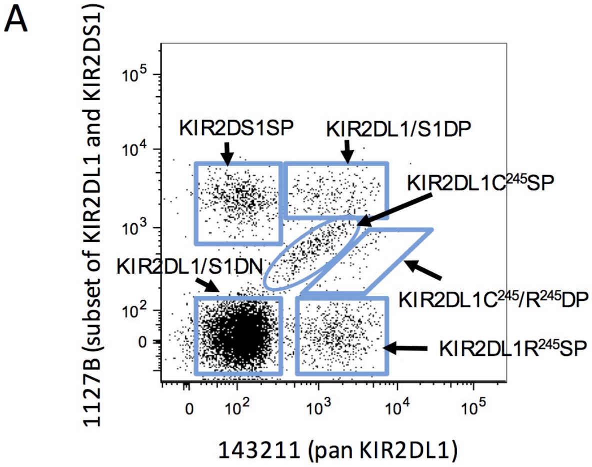

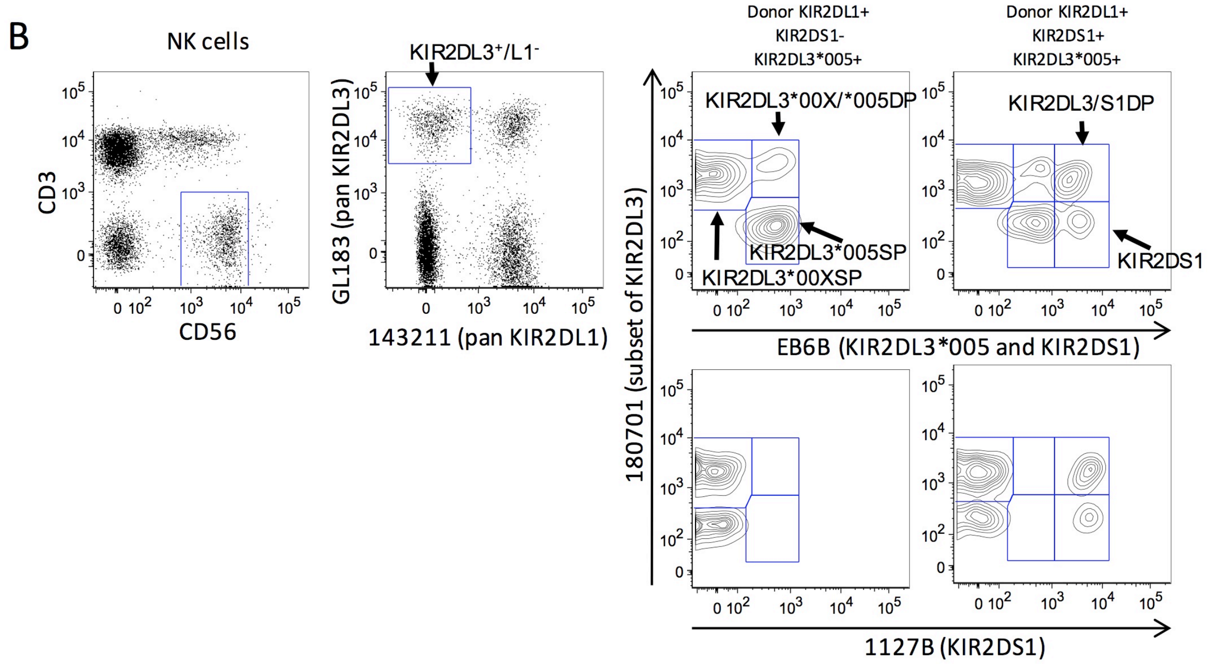

Supplemental Figure 3: Flow cytometric gating strategy and evaluation of KIR+ NK populations. (**A**) Flow cytometry gating strategy of distinct NK populations. (**B**) CD107a mobilization in KIR2DL2/L3/S2^{+}NKG2A^{-}2DL1/S1^{-} 3DL1^{-} cells following co-culture with 721.221 and 721.221-HLA-Cw4 (721.221-C2) target cells without additional antibody or in the presence of 1127B or 11PB6 antibody. Results are shown from four different donors with KIR ligands as indicated, representative of ten donors tested.

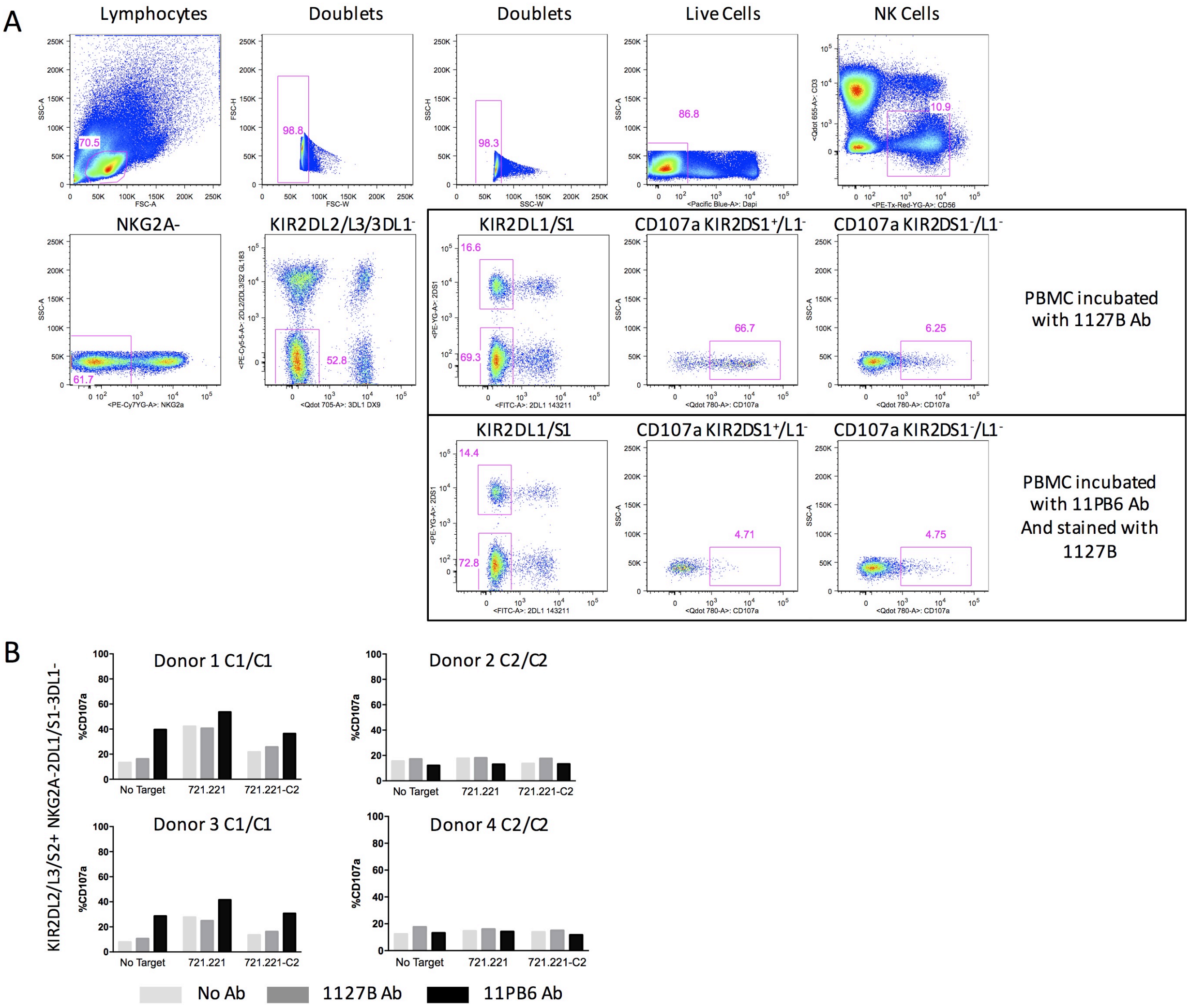

Supplemental Figure 4: Titration of monoclonal antibody 1127B. (**A**) Cytotoxic response, as measured by CD107a mobilization, of KIR2DS1sp NK cells incubated 3 hours with monoclonal antibody 1127B. (**B**) Flow cytometry staining with 1127B mAb during the CD107a mobilization assay.

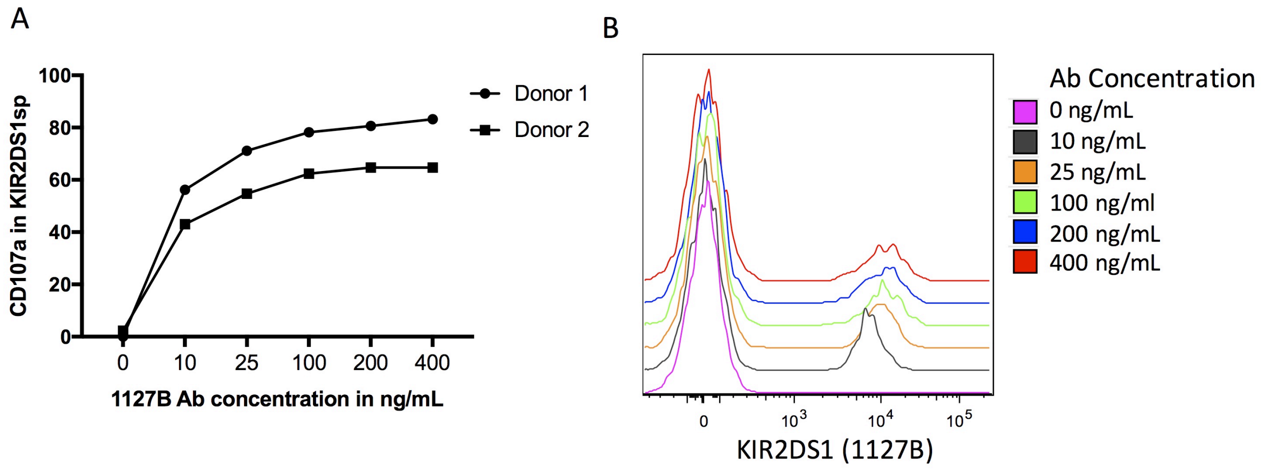

Supplemental Figure 5: Inhibition and cell surface expression of KIR2DL1 allotypes. NK effector response and inhibition of NK cells expressing KIR2DL1-C^{245} vs KIR2DL1-R^{245} allotypes from **(A)** HLA-C1/C1 **(B)** or -C1/C2 donors upon co-incubation with the HLA class I-negative target cells K562 and 721.221 or with 721.221 transfected with HLA-C1 or -C2 for 5 hours (upper graphs) or 24 hours (lower graphs). **(C)** Cell surface detection of KIR2DL1 allotypes by flow cytometry following ligand engagement.

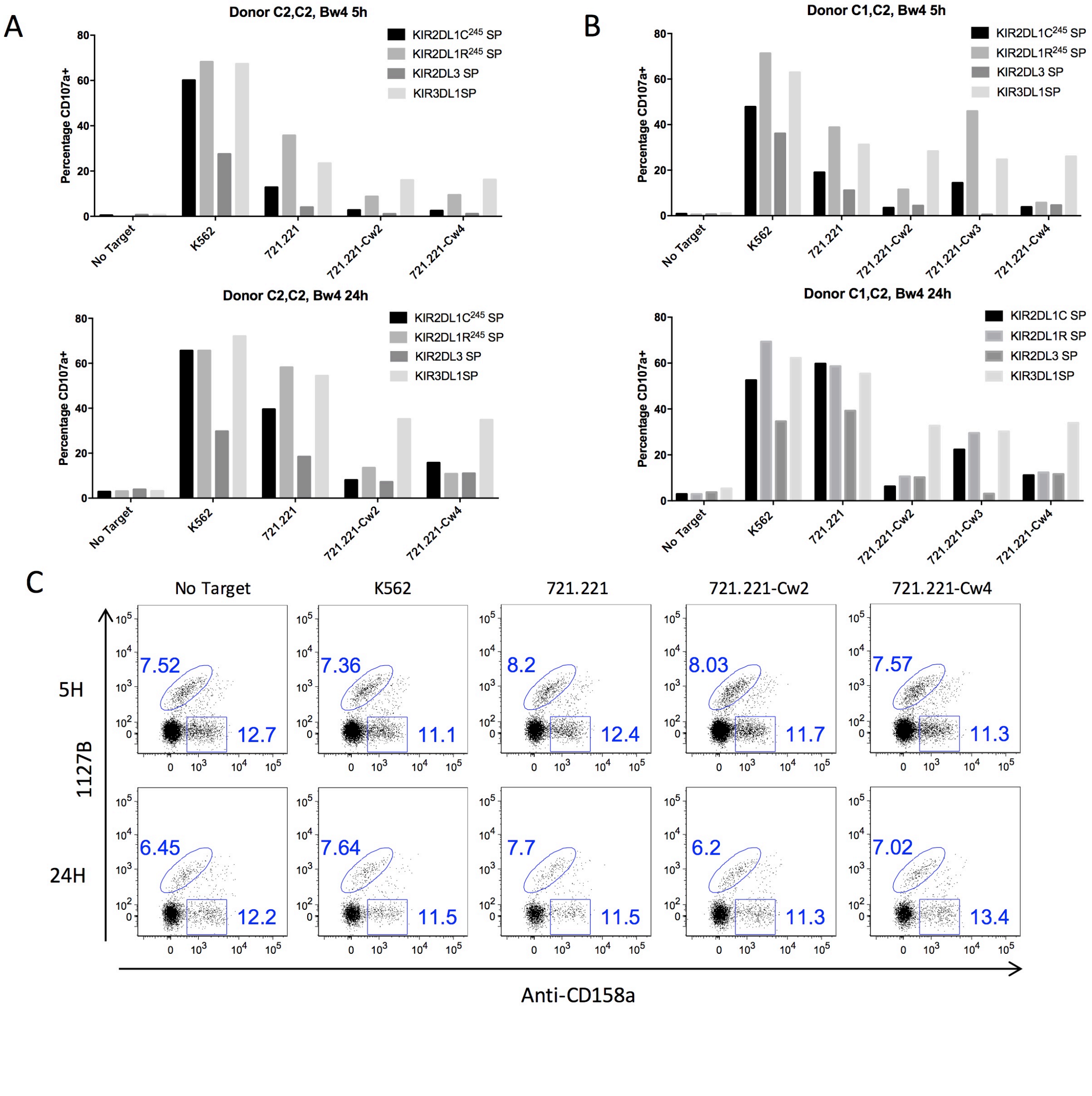

Supplemental Figure 6: Impact of co-expression of educated receptors on the education of KIR2DS1. Functional assays represent CD107a staining on different subpopulations of NK cells single positive, double positive or double negative for KIR2DL1 and KIR2DS1. PBMC from nineteen donors (6 *HLA-C1/C1*, 7 *HLA-C1/C2* and 6 *HLA-C2/C2*) were stimulated for 5 hours with 721.221 or 721.221-C2 target cells. (**A**) Responsiveness of NKG2A$^-$ KIR2DL2/L3/S2$^-$ 3DL1$^-$ NK cells. (**B**) Responsiveness of NKG2A$^+$ KIR2DL2/L3/S2$^-$ 3DL1$^-$ NK cells. Unpaired Mann-Whitney test was used. Symbols represent individual samples (mean ± SEM). * *p<0.05,* ** *p<0.01,* *** *p<0.001,* **** *p<0.0001.*

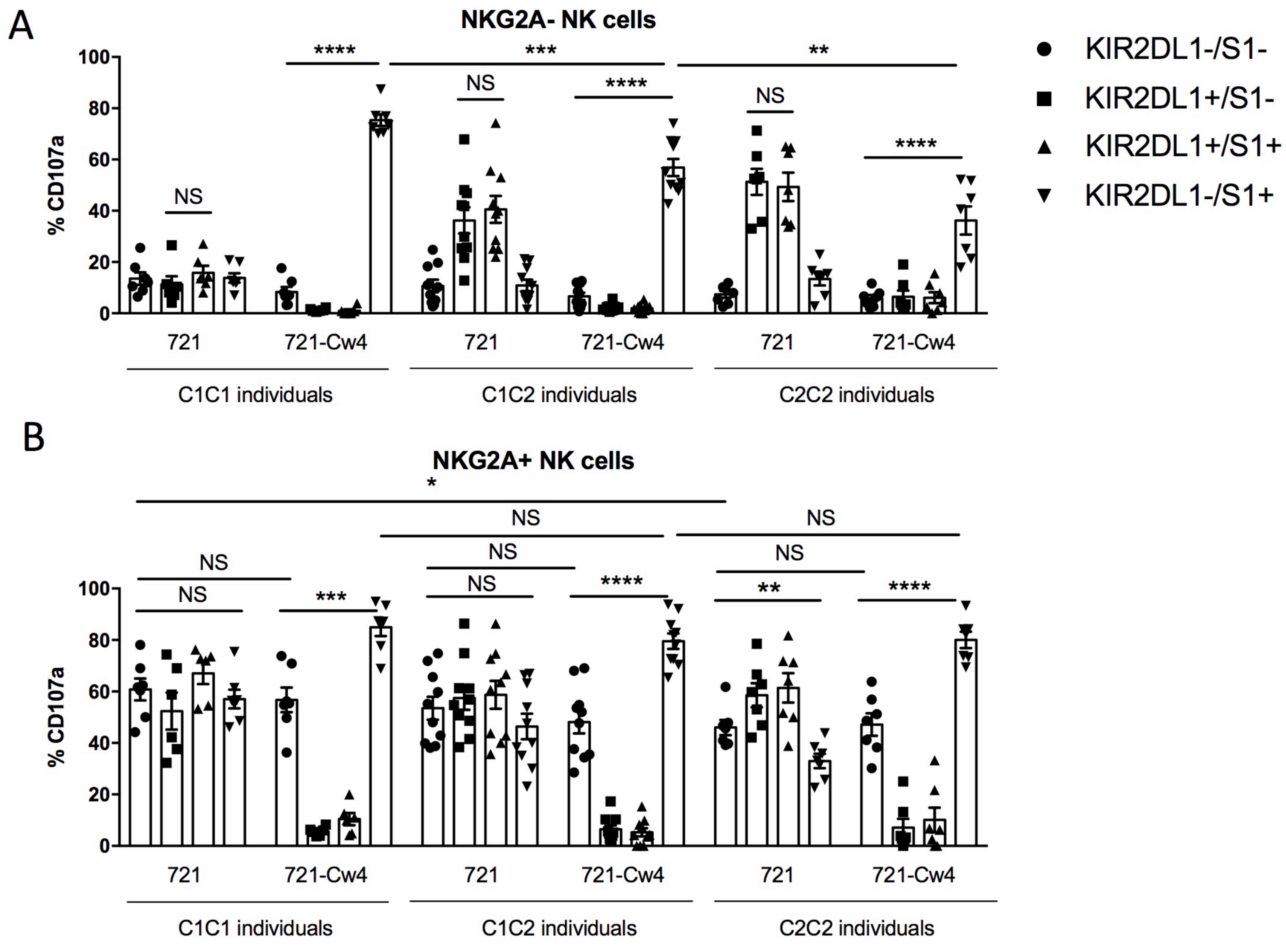

Supplement: Supplementary file 1 [file Data_Sheet_1.pdf]
